# Delayed Diagnosis of Non-ST Segment Elevation Myocardial Infarction in a Young Patient with Multivessel Disease and Familial Hypercholesterolemia Complicated by Cardiogenic Shock Finally Treated with Intra-Aortic Balloon Pump as a Bridge to Extra Corporeal Membrane Oxygenation

**DOI:** 10.1155/2019/9470131

**Published:** 2019-01-17

**Authors:** Rafał Januszek, Magdalena Jędrychowska, Piotr Jankowski, Dariusz Dudek, Stanisław Bartuś

**Affiliations:** ^1^2^nd^ Department of Cardiology and Cardiovascular Interventions, University Hospital, Krakow, Poland; ^2^1^st^ Department of Cardiology, Jagiellonian University Medical College, Krakow, Poland; ^3^Department of Interventional Cardiology, Jagiellonian University Medical College, Krakow, Poland

## Abstract

Delayed diagnosis of coronary artery disease in young patients after cardiac arrest of unknown origin could increase the risk of death in further diagnostic and therapeutic process. Familial history of premature coronary atherosclerosis and hypercholesterolemia could help in proper diagnosis and treatment. We present a case of a 29-year-old female admitted to the catheterization laboratory with cardiogenic shock and multivessel coronary artery disease treated successfully with multivessel percutaneous coronary intervention and intra-aortic balloon counterpulsation as a bridge to extracorporeal membrane oxygenation.

## 1. Introduction

Familial hypercholesterolemia (FH) is one of the most common causes of premature coronary atherosclerosis and, in consequence, major cardiovascular events but still very often remains unrecognized early enough and therefore is not treated properly. The risk of coronary heart disease in patients with FH is estimated to be increased at least 10-fold [1]. Based on the presented case of a young woman being treated at our department due to acute myocardial infarction (MI) complicated by cardiogenic shock (CS), we would like to draw attention to several recently discussed problems in cardiology: firstly, severe cardiovascular complications due to undiagnosed FH at a young age; secondly, the cardiac diagnostic process of patients after sudden cardiac arrest; thirdly, the issue of noninfarct related artery (IRA) revascularization in acute MI; and finally, the role of intra-aortic balloon counterpulsation (IABP) in CS as a bridge to extracorporeal membrane oxygenation (ECMO).

## 2. Case Presentation

**Figure 1:**
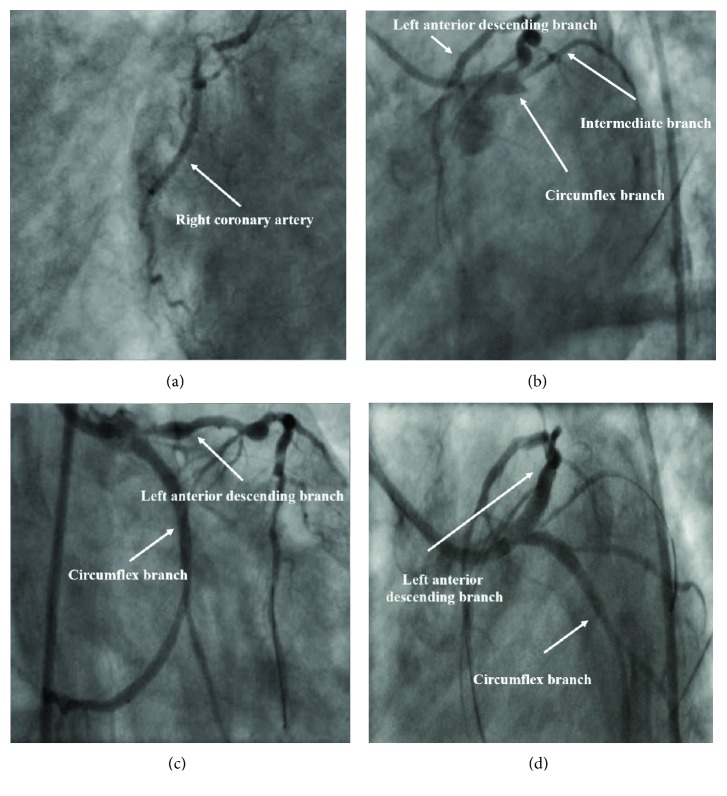
(a) Coronary artery angiography: right coronary artery. (b) Coronary artery angiography: left coronary artery. (c) Coronary angiography after percutaneous coronary intervention within circumflex branch. (d) Coronary angiography after percutaneous coronary intervention within the left anterior descending branch.

A 29-year-old female was admitted to the emergency department (ED) after sudden cardiac arrest at home followed by effective resuscitation. The first recorded heart rhythm was ventricular fibrillation. After effective resuscitation, due to respiratory insufficiency, the patient was intubated and mechanical ventilation was set. Electrocardiography revealed ST-segment depressions up to 1 mm in I, aVL, II, III, and V1-4 leads. The patient was then transported to the ED. At admission to the ED, the patient was hemodynamically stable and preserved systolic blood pressure without inotropes. Considering blood tests, including elevated level of serum D-dimers, at first, computed tomography of the head and chest in pulmonary embolism algorithm was performed. There were signs of cerebral stroke and evident pulmonary embolism. Chest radiograph depicted features of pulmonary edema. At that time, a cardiological consultation was made on the basis of which the cardiac echocardiography was ordered at patient's bedside. Cardiologist consultation and echocardiography revealed impaired left ventricle ejection fraction (LVEF) (approx. 25-30%) with regional contractility impairment (recent akinesis of the lateral, inferior, and posterior walls and hypokinesis of other walls) and moderate mitral regurgitation. The patient was immediately qualified for coronary artery angiography and transferred from ED to the catheterization laboratory (CathLab). During transportation to the CathLab, the first symptoms of CS had developed, and the patient was given first inotrope—noradrenaline. Coronary artery angiography revealed multivessel disease including recessive right coronary artery with 60% stenosis; the left main coronary artery was without significant stenoses, ostial occlusion of the dominant circumflex branch (Cx), left anterior descendent artery (LAD) with multiple significant stenoses: ostial: 80-90%, proximal segment: 70%, medial segment: 80%, and distal segment: 60%, the intermediate branch with 80-90% ostial stenosis. This is presented in Figures 1(a) and 1(b). Percutaneous coronary intervention (PCI) within Cx with thrombectomy and drug-eluting stent (DES) implantation, Onyx 3.5 × 12 mm, 18 atm., was performed and followed by sequential inflation in the intermediate branch (c.b. 1.5 × 12 mm, 20 atm.) and Cx (4.0 × 8 mm, 24 atm.). Due to the progression of CS and no improvement after adding other inotropes (dopamine, dobutamine) at high doses, the decision was made to implant IABP and perform PCI of LAD. Predilation with b.c. 3.0 × 15 mm 20 atm. was performed. Implantation of two DES in the proximal and medial segment was performed: DES Ultimaster (4.0 × 18 mm, 18 atm.) and DES Create (3.0 × 31 mm, 16 atm.). Postdilatation, with b.c. 4.0 × 15 mm, up to 20 atm., was executed using the proximal optimization technique. Then, the patient was then sent to the intensive care cardiology unit in a critical state, with the blood pressure of 40-50 mmHg. Control echocardiography revealed decreased LVEF (around 20%). A fourth inotrope was added (adrenaline) without significant improvement of systolic blood pressure. After consultation at the cardiac surgery department, the patient was qualified for ECMO treatment. The patient was successfully surgically connected to ECMO after transportation to the cardiac surgery unit and surgeons were able to wean from the ECMO, due to the improvement of LVEF after five days. During the long stay at the cardiac surgery unit, several complications developed, including severe bleeding demanding transfusion of more than 16 units of blood products, acute ischemia of the left lower limb, left femoral artery thrombectomy and surgical angioplasty, respiratory tract infections with negative culture (bronchoscopy), pneumonia, pulmonary edema, and recurrent mechanical ventilation necessity. The patient finally survived, was weaned from mechanical ventilation, and was discharged for further rehabilitation after almost 70 days without any neurological deficiencies and able to walk using a walker.

## 3. Discussion

According to the lack of any previous diagnostic results indicating FH, making the diagnosis at admission was difficult. Correctly collected interviews by rescuers during resuscitation of the patient at her home could shorten the time of transportation to CathLab. The patient's sister's MI performed at a young age and her father's death as a complication of acute MI indicated suspicion of an inherited issue in our patient. All patients following the return of spontaneous circulation who do not have an alternate explanation for cardiac arrest irrespective of the electrocardiography findings may be considered for coronary angiography and possible PCI [2]. It has been demonstrated that direct transport to CathLab was associated with a significant increase in survival rate [3]. Also, the bedside echocardiography should be performed immediately if there is suspicion of underlying cardiac ischemia [4]. Delayed time of reperfusion in MI patients is especially important because it has great impact on mortality rate [5]. In the current case, there was an immediate worsening of clinical state after PCI of IRA at the CathLab. Then, the IABP was inserted and a decision was made to perform non-IRA PCI. The PCI of the IRA and complete revascularization approach were compared in four randomized clinical trials: PRAMI, CvLPRIT, DANAMI-3–PRIMULTI, and Compare-Acute trial. None of them has demonstrated superiority of complete revascularization in mortality reducing to primary PCI of the IRA [6–9]. Another option of left ventricle support device for that patient could be the Impella heart pump. However, first, we did not have this device in CathLab at that time, and secondly, the device is preferred in stable patients with low LVEF undergoing PCIs, although it is also successfully used in acute conditions. Therefore, according to the ESC 2017 consensus, non-IRA PCI during the index procedure is rather not advised in patients with CS [10]. However, in this particular case, we decided to perform PCI of non-IRA due to the young age of the patient. Cardiogenic shock is the main cause of death related to acute MI, with a mortality rate of 40-50% in the absence of aggressive and highly specialized technical care [11–13]. Until 2012, the European and American consensus considered the use of IABP; in the case of post-AMI, cardiogenic shock was rather recommended. Based on the results of recent meta-analyses, the current consensus has changed its recommendations, and now, IABP should be considered in acute MI-related CS and mechanical complications instead of short-term percutaneous mechanical support which is preferred in these patients [3, 14]. Currently, the European consensus does not advocate for the routine usage of IABP due to the results of the large randomized multicenter IABP-SHOCK II trial which showed no survival benefit with IABP support. At 30 days, there was no significant difference in the primary outcome of mortality between IABP and control groups [15]. However, future longer follow-up could modify this approach. On the other hand, the specific use of IABP as a bridge to ECMO in CS has not been closely evaluated. Such a solution may bring special benefits at centers without a cardiac surgery department, without regular access to short-term left ventricle support devices. In this case, additional hemodynamic support is necessary to secure the patient during the time of transportation. In this specific situation, the lack of IABP affects the time for hemodynamic stabilization, length of stay at the ICU, the dose and duration of catecholamine therapy, and renal function. Our main purpose was to maintain the minimal critical organ blood supply and oxygenation until transportation to the cardiac surgery center and then connect the patient to ECMO. It should be noticed that despite the persistent extremely low systolic blood pressure and delay associated with the necessity of organization and transportation to another center, our patient does not have any permanent neurological deficiencies.

## 4. Conclusions

In conclusion, IABP could serve as bridge therapy to ECMO in young and promising patients with acute MI in the course of multivessel disease complicated by CS. Nationwide screening programs for familial hypercholesterolemia should be conducted to detect patients with FH at the youngest possible age and treat them properly. Young patients after sudden cardiac arrest, and in particular with innate risk factors for premature atherosclerosis, should be first consulted at CathLab to implement the appropriate diagnostic and treatment procedures, which could affect the overall hospital stay length and condition of the patient. Selected patients with cardiogenic shock may benefit from PCI of non-IRA.
